# Supplementary material for: Telomere-to-telomere genome sequence of the model mould pathogen Aspergillus fumigatus
Source: Nat Commun. 2022 Sep 14;13:5394. doi: 10.1038/s41467-022-32924-7 (PMC9472742; doi:10.1038/s41467-022-32924-7)
Supplement: Supplementary file 2 — Description of Additional Supplementary Files [file 41467_2022_32924_MOESM2_ESM.pdf]

Title: Supplementary Data 1:
Description: Assembly metrics for Pacific Biosciences, Oxford nanopore and Illumina HiSeq data

Title: Supplementary Data 2:
Description: Variation between CEA10 and A1160. 130 variants with predicted high, moderate or low impact on gene function are listed from 395 supported variants in the comparison

Title: Supplementary Data 3:
Description: NCBI SRA accession numbers

Title: Supplementary Data 4:
Description: A. fumigatus genome assemblies obtained from NCBI assembly database (https://www.ncbi.nlm.nih.gov/assembly) and used to analyse CEA10 and Af293 translocation breakpoints

Title: Supplementary Data 5:
Description: Translocation breakpoint flanking sequences from Af293 and CEA10
