# Supplementary material for: Telomere-to-telomere genome sequence of the model mould pathogen Aspergillus fumigatus
Source: Nat Commun. 2022 Sep 14;13:5394. doi: 10.1038/s41467-022-32924-7 (PMC9472742; doi:10.1038/s41467-022-32924-7)
Supplement: Supplementary file 7 — Supplementary Data 5 [file 41467_2022_32924_MOESM7_ESM.docx]

**Supplementary Data 5: Translocation breakpoint flanking sequences from Af293 and CEA10.**

Refer to Fig. 5 for details

**BP1**

Af293-Chr1-BP1

Af293-Chr1-492222

>Chr1

CTTCTCGTATGATTGCTATTTGCTCCTTATCCATGGCTTGGCCTCTAGATGAAGATTCCCGCACATCACCCATGAAGAATCACCGTAAAGCACAGAATAGTCGAGGCATCTGCCAGACTCGACCTGTAGCTGCACCATAAAGCAGTGGTTGCCTCGGGCAATGTGATTGTGAAACCCTTGCCACCACCACCACCACCACCCCCCATTCCCTCGACTGGTCTCGTAGTCTGGTATTCACCAAAGTTCTTTGCATTCGAGATATAAGGTTACAATACGAGAATAGGCTGCAGGATAAGCCACAGGAAGCGCCCCGCGGAGGGCGGGAATTGGGGCCCCGATAATAGTGGCCTTGGACCGTCTCCACCACGATCCACAACAATCATAGATAGTGCTGGACACTCGCACCGACCCCTCGACACT

**BP2**

>CEA10-Chr1 BP2

GAGTGTCCATCCGTACGAGATGCGGCTTTGGCGCTGTGGCGGCTAATTATTTGGACTTCGTGTTGGTCAGTAATTTACTTGCATGTTAGCTTGTTTGAAGGCGACTAGTGTCCTTGACATGGTTGGATTGAAACACTGGTTGAGACAGAGTTGATCGAGACGGGATTCCGATACATCCAAGTGGAT

**CEA10chr1>>>>534357**

CGTAACCAGTAACCCATGCCCCACTCACTTTGGGGCTCCGTGCGCCCAATCATCTCAGCTTT

**1473023<<<<Af293chr8**

GGTCGTCAGCGCCGTATCAGGTGGTGCTTGCTTCCCAGTTTATATTAGCCTTCAAAGTTTGGCCCTGATCAACACAGCTTTTTTGTCCACAGGCTAGGACATGGCCGAACTGGCGAAACGCCAGTTTCGCACCAATTTCGCTCAAACAGTGTG

**BP3**

CEA10 Chr6 3645813

>CEA10-chr6-BP3

GGGATCTGAGGAGTCAGTCCTATGTTGCTTCCACGTTGTCCTATTTGTTTGGCGCTCCTCCCATTTGCCATTTTGTGCTCCTTCGGAGGGAAAAGCGACACTTAAGTCGATATCCGGATCGTCGCTGACCCAGGACTGGAGCTTATTCGAGGTCCTTCGGTTGAGCTTTGCTGTATCTGTTTTCAGTCGTTGGAATTGTAAAGCGAGGACCTGTGAATTCCCTCTTGTGGTTAGTTTTGCCGTTTTGGGTATGGTCGCCCGATACGCCGTCTTGTAAACACCGCCCCCATCGTGACCTCCAGTATATAAGAACCGTCCCCTCGCTGTTCTAACCATCCTCTCTCCCAACTTCTTCTTTTCCTCCCAACTCCCCCAAATATTCTACACCCTTCCGGTCGCTGCC

**BP4**

>AF293-Chr6-BP4

TGAAGAAACGTTGACTTCAGATCTCTCCCAAAGAATAGAGGAGAAAGCGCCGTTTGATTTGGGCCACGAAGGAAACTAACTATAACTTCTCAGCCTGTCGTTAATAGAACTTCCAAACACTTAGACTTCAATCACAGGGCCATTCAACGTCAGCACAGAGTAGACAAGAAGTGGATAGGTAAAACT

**CEA10chr6>>>3239542**

ACAAAGGATAGGGCGGGTTACTGGTTACGATCCACTTGGATGTATCGGAATCCCGTCTCGAT

**534357<<<<<CEA10chr1**

CAACTCTGTCTCAACCAGTGTTTCAATCCAACCATGTCAAGGACACTAGTCGCCTTCAAACAAGCTAACATGCAAGTAAATTACTGACCAACACGAAGTCCAAATAATTAGCCGCCACAGCGCCAAAGCCGCATCTCGTACGGATGGACACTCCA

**BP5**

>CEA10-Chr6-BP5

ATGAAGAAACGTTGACTTCAGATCTCTCCCAAAGAATAGAGGAGAAAGCGCCGTTTGATTTGGGCCACGAAGGAAACTAACTATAACTTCTCAGCCTGTCGTTAATAGAACTTCCAAACACTTAGACTTCAATCACAGGGCCATTCAACGTCAGCACAGAGTAGACAAGAAGTGGATAGGTAAAAC

**>>>CEA10chr6 3605715**

TACAAAGGATAGGGCTCATTCAAGCTATTTAAATCATCTGTCACCCCACATTTTGCATCGCC

**1473027>>>> Af293chr8**

TTTGTACGCATTTTGCATTGCCTTTGTATGCATTTTGCATCGCCCTTGTACGCATTTTGCATCGTTTTCGTACTCGTTTTGCATCGCTTTTGTATTCACTTTGCATCCATTTTGTATCCCTTTTCAGTCCACACCCAACTCGTTCGCTCCTCA

**BP6**

CEA10 chr6 3645806

>CEA10_Chr6_BP6

GAATGTAGGGATCTGAGGAGTCAGTCCTATGTTGCTTCCACGTTGTCCTATTTGTTTGGCGCTCCTCCCATTTGCCATTTTGTGCTCCTTCGGAGGGAAAAGCGACACTTAAGTCGATATCCGGATCGTCGCTGACCCAGGACTGGAGCTTATTCGAGGTCCTTCGGTTGAGCTTTGCTGTATCTGTTTTCAGTCGTTGGAATTGTAAAGCGAGGACCTGTGAATTCCCTCTTGTGGTTAGTTTTGCCGTTTTGGGTATGGTCGCCCGATACGCCGTCTTGTAAACACCGCCCCCATCGTGACCTCCAGTATATAAGAACCGTCCCCTCGCTGTTCTAACCATCCTCTCTCCCAACTTCTTCTTTTCCTCCCAACTCCCCCAAATATTCTACACCCTTCCGG

**BP7**

CEA10_Chr6_3698870

>CEA10_Chr6_BP7

GCGAGTTCTCGCTGAGGATTGTCTCCTCAGGACGGTGACTAAAACAATGCTTGGCGGGTGCAGAATAGTGGTGTGTAGCACGTGCTTGAGTGCTAAAGCGGCGGCTCGCAGAGGGAAAATACAGGTGGAAATGCGGCCGTTTATGGCCTCAGTAGCTGTCATTTTCGGCGTCGATTTCACAGGTCCCCATATTCTAGTTGTAATATTCGATTACTCCTGGGAAAAGGAGTCAGGGATGAGTGAAATCAAATCGGCCCCTGCATTTCTCTCGATCGCCGTCTTAAATATTTGCATCGGCTCTTAATTACGACCCACGGCGTCGGTACAGGCCAAGGAGGTAAGTGTTTCCGAAGTATAGCAGTATCATCCATGGTCATTTAGAAGTAAATGGGCTCCAAAGT

**BP8**

CEA10 Chr6 3724525

>CEA10_Chr6_BP8

AAAATGCCTCAAGTTGGCTCGAGTCAACAAGGCGTCAAAAGGGTGGGAAAGCAGCGACGAGAGTTGCCCATCATCTGGAGACGGACGAGCAGGCCATCTTATAGACCACGCACCGAGATGCCAAGCCTCAACCACGCGCTTTCCCCAAATAGGAACGCTATACCGCTTCTAGTAATTCGCCGTCACCGCAGACTACGGAGTAGCGAAGCACTCTGCTCTCCGCGTAGGAGACCAGTCGAGGGAATGGGGGGGGGTGGTGGTGCCAAGGGTTTCACAATCACATTGCCCGAGGCAACCACTGCTTTATGGTGCAGCTACAGGTCGAGTCTGGCAGATGCCTCGACTATTCTGTGCTTTACGGTGATTCTTCATGGGTGATGTGCGGGAATCTTCATCTAGAGG

**BP9**

Af293-chr8-BP9

Af293 Chr8-1519051

>Chr8

ATGTAGGGATCTGAGGAGTCAGTCCTATGTTGCTTCCACGTTGTCCTATTTGTTTGGCGCTCCTCCCATTTGCCATTTTGTGCTCCTTCGGAGGGAAAAGCGACACTTAAGTCGATATCCGGATCGTCGCTGACCCAGGACTGGAGCTTATTCGAGGTCCTTCGGTTGAGCTTTGCTGTATCTGTTTTCAGTCGTTGGAATTGTAATATTCGATTACTCCTGGGAAAAGGAGTCAGGGATGAGTGAAATCAAATCGGCCCCTGCATTTCTCTCGATCGCCGTCTTAAATATTTGCATCGGCTCTTAATTACGACCCACGGCGTCGGTACAGGCCAAGGAGGTAAGTGTTTCCGAAGTATAGCAGTATCATCCATGGTCATTTAGAAGTAAATGGGCTCCAA

**BP10**

AF293_Chr8_1519051

>AF293_Chr8_BP10

ATGTAGGGATCTGAGGAGTCAGTCCTATGTTGCTTCCACGTTGTCCTATTTGTTTGGCGCTCCTCCCATTTGCCATTTTGTGCTCCTTCGGAGGGAAAAGCGACACTTAAGTCGATATCCGGATCGTCGCTGACCCAGGACTGGAGCTTATTCGAGGTCCTTCGGTTGAGCTTTGCTGTATCTGTTTTCAGTCGTTGGAATTGTAATATTCGATTACTCCTGGGAAAAGGAGTCAGGGATGAGTGAAATCAAATCGGCCCCTGCATTTCTCTCGATCGCCGTCTTAAATATTTGCATCGGCTCTTAATTACGACCCACGGCGTCGGTACAGGCCAAGGAGGTAAGTGTTTCCGAAGTATAGCAGTATCATCCATGGTCATTTAGAAGTAAATGGGCTCCAA

**BP11**

Af293_Chr8_1544700

>Af293_Chr8_BP11

CCAATGGCAAAAATGCCTCAAGTTGGCTCGAGTCAACAAGGCGTCAAAAGGGTGGGAAAGCAGCGACGAGAGTTGCCCATCATCTGGAGACGGACGAGCAGGCCATCTTATAGACCACGCACCGAGATGCCAAGCCTCAACCACGCGCTTTCCCCAAATAGGAACGCTATACCGCTTCTAGTAATTCGCCGTCACCGCAGACTACGGAGTAGACTGAAAAGGTGTCCGGACGATTCCTATCAGTCCAGGGTCTCTATTTATTCTTCAACACAAGACTCTAGTGCCCGCGGCCCGCCTACCCCTAACTAAGGGGGCTAGCGCCTGCATATTGTAAAGCCATCTAATGCCCCGGTAGCCGAAAAGAGTATGTTGCATGCATATCGTATCAGCTGCTGTAATAA

**BP12**

CEA10_Chr8_1459118

>CEA10_Chr8_BP12

TGGCACATTTTGTGAACGAAGCTACTAATGCTCAATCCGATCATAGTCTAGTAATATCTGGTCATCCTTCCACTTACCTATTGTGTAGGACATTTAATCTCACTCGTCTTCTAGGGGCTAGATTGTATTGATACATTGGTTACCACCACTAGCTCCTAGGCTGATAACAAGATATTGAAATAGAATACCACTACGGAGTAGACTGAAAAGGTGTCCGGACGATTCCTATCAGCCCAGGGTCTCTATTTATTCTTCAACACAAGACTCTAGTGCCCGCGGCCCGCATACCCCTAACTAAGGGGGCTAGCGCCTGCATATTGTAAAGCCATCTAATGCCCCGGTAGCCGAAAAGAGTATGTTGCATGCATATCGTATCAGCTGCTGTAATAACAACAGGGG
